# Rhein promotes the proliferation of keratinocytes by targeting oestrogen receptors for skin ulcer treatment

**DOI:** 10.1186/s12906-022-03691-1

**Published:** 2022-08-05

**Authors:** Ning Xu, Yuanran Chen, Dongjie Guo, Yu Deng, Wanjun Guo, Xin Liu, Yi Wang, Hanzhi Lu, Aijun Liu, Jianyong Zhu, Fulun Li

**Affiliations:** 1 Department of Dermatology, Yueyang Hospital of Integrated Traditional Chinese and Western Medicine, Shanghai University of Traditional Chinese Medicine, Shanghai, 200437 China; 2 School of Medicine, Chengdu University, Chengdu, 610000 China; 3 Department of Pharmacy Research, Yueyang Hospital of Integrated Traditional Chinese and Western Medicine, Shanghai University of Traditional Chinese Medicine, Shanghai, 200437 China

**Keywords:** Rhein, Oestrogen receptor, Wound healing, Skin ulcer FosB, JunD

## Abstract

**Background:**

The Sheng-ji Hua-yu (SJHY) formula is a quite effective Traditional Chinese Medicines (TCM) in the treatment of delayed diabetic wounds. Previous research has shown that the SJHY formula has significant anti-inflammatory and wound-healing effects, but the precise mechanism remains unknown. The purpose of this study was to evaluate the effects of rhein, a compound extracted from SJHY formula, in keratinocytes and to investigate the underlying mechanisms.

**Methods:**

Microscale thermophoresis (MST) technology was used to confirm that rhein binds directly to oestrogen receptors (ERs). Rhein was then used to treat keratinocytes in vitro. Cell cycle and proliferation analysis, Real-time polymerase chain reaction (RT-PCR) and Western-blot were conducted.

**Results:**

Rhein increased the proportion of cells in the S phase of the cell cycle and promoted keratinocyte proliferation. ICI 182,780, an ER inhibitor, was also used to treat keratinocytes. The expression of c-myc mRNA and protein induced by rhein was antagonized by ICI 182,780, indicating that this induction is ER dependent. Intervention with ICI 182,780 had no effect on the upregulation of FosB and JunD, indicating that activator protein 1 (AP-1) members (FosB and JunD) are involved in rhein-induced c-myc mRNA and protein expression but does not require the ER.

**Conclusion:**

The present study found that rhein stimulates keratinocyte proliferation by activating the oestrogen signalling pathway via the oestrogen receptor, which induces the expression of c-myc in collaboration with FosB and JunD, thereby accelerating the process of re-epithelialization.

**Supplementary Information:**

The online version contains supplementary material available at 10.1186/s12906-022-03691-1.

## Background

Skin ulcers are the most common complication among patients with skin diseases caused by various factors. Bacterial infection, trauma, radiation, psychological spirit, immune deficiency, and other factors are common pathogenic factors of skin ulcers [1]. With multiple initiating factors of skin ulcers, the complicated mechanism involved in wound healing remains unclarified, which greatly restricts the further development of effective treatment methods and agents. Pain, wound contamination, or even amputation followed by delayed closure has left a heavy burden on skin ulcers patients. Regular first-line therapies, such as debridement, dressing applications and anti-microbial agents do not always reach a satisfying endpoint [2]. So, complementary and alternative medicine like Traditional Chinese Medicine (TCM) may be meaningful in promoting the chronic wounds.

Estrogen, a hormone secreted primarily by the ovaries, has been reported to be involved in several pathophysiological processes such as neoplasia [3], pain modulation [4], and osteoporosis [5]. In general, estrogen exerts its effects by interacting with two distinct receptor subtypes, Estrogen Receptor (ER) α and Erβ [6]. Some studies revealed that oestrogen signaling pathways can promote wound healing by enhancing keratinocyte migration [7, 8], fibroblast migration [7] and angiogenesis [8]. But at the same time, some natural products, which was called phytoestrogens, may play a similar role as endogenous estrogens. Rhein is a natural anthraquinone derivative derived from the rhizomes of several TCM herbs, including *Rheum palmatum* L. (also known as “da huang”) and *Polygonum multiflorum* (also known as “he shou wu”). Some evidences indicated that rhein intake could be a risk factor in some estrogen-dependent diseases [9, 10]. But on the other hand, rhein was reported to boost wound-healing via potential anti-bacterial and anti-inflammation activities. It has been shown that rhein inhibits the proliferation of keratinocytes colo-16 (a squamous carcinoma cell line) by blocking the cell cycle in the G_1_ phase, while our study showed that rhein induces the proliferation of keratinocytes HaCaT by increasing the proportion of cells in the S phase of the cell cycle, which suggests that rhein may have a bidirectional regulatory effect on keratinocytes proliferation. Therefore, rhein, as an effective active ingredient of Chinese medicine rhubarb, has great potential for development and clinical application in the field of skin diseases. Based on previous researches, we aim to discuss whether rhein improves wound-healing process by upregulating oestrogen signaling pathway.

In this study, we investigated the cell viability and proliferation of keratinocyte (HaCaT) treated with rhein and dissected the underlying mechanisms. We used microscale thermophoresis (MST) technique to verify the direct binding ability of rhein to the target protein (estrogen receptor) and to verify whether rhein activates estrogen signaling pathway through estrogen receptor and induces c-myc expression together with AP-1 members (FosB, JunD). The present study will help to clarify the potential therapeutic significance of rhein for skin ulcers therapy.

## Materials and methods

### Microscale thermophoresis assay

Microscale Thermophoresis Assay (MST) was used to determine the binding affinity of ERs for rhein. Recombinant ERα (Thermo Fisher Scientific, U.S.), ERβ (Thermo Fisher Scientific Inc, U.S.) and Rhein (National Institutes for Food and Drug Control, China) were labelled with fluorescence dye using Protein Labeling Kits (NanoTemper Technologies, Germany). The assays were performed in a buffer (pH 7.4) containing 20 mM Tris, 0.3 M NaCl, 5% glycerol, 3% Dimethylsulfoxide (DMSO), and 0.05% Tween-20. After a 30-min incubation, the samples were loaded into standard glass capillaries for Monolith NT.115 system (NanoTemper Technologies, Germany). During the MST experiments, the concentration of the labelled ERs was kept constantly at 6.74 μM, while the concentration of rhein was serially diluted at a ratio of 1:2. In total, 16 titration series of rhein from the maximal final concentration of 0.1 mM to the minimal concentration of 3.05 nM were prepared and mixed with the labelled ERs. Fluorescence was analysed in the Monolith NT.115 instrument. The MST power and excitation power used were 20% and 75%, respectively.

### Cell culture

Human Immortalized Keratinocyte (HaCaT) cells were obtained from Ruijin Hospital, Shanghai Jiaotong University School of Medicine (Shanghai, China). HaCaT cells were cultured in dulbecco's modified eagle medium (DMEM) (Gibco, U.S.) supplemented with 10% foetal bovine serum (Gemini Biosciences Ltd, U.S.) and 1% solution of penicillin and streptomycin (Thermo Fisher Scientific Inc, U.S.). All cells were incubated at 37 °C in a 5% CO_2_ humidified incubator (Thermo Fisher Scientific Inc, U.S.). Cells passaged under oestrogen-free culture conditions did not exceed 4 generations. HaCaT cells were obtained from Ruijin Hospital, Shanghai Jiaotong University School of Medicine (Shanghai, China). HaCaT cells were cultured in DMEM (Gibco, USA) supplemented with 10% foetal bovine serum (Gemini, USA) and 1% antibiotics (Thermo Fisher Scientific, USA). All cells were incubated at 37 °C in a 5% CO_2_ humidified incubator. Cells passaged under oestrogen-free culture conditions did not exceed 4 generations.

### Cell viability assay

HaCaT cells were seeded into 96-well plates (5 × 10^3^ cells/well) to allow attachment, incubated overnight, and then treated with phenol-free DMEM with various concentrations of rhein (6.25, 12.5, 25, 50, 100 μM) for 24 h. Next, 10 *μ*L of CCK-8 solution (Bimake, USA) was added to each well, and the plates were incubated for an additional 70 min at 37 °C in a 5% CO_2_ incubator. The absorbance at 450 nm was measured using a spectrophotometer (Tecan, Switzerland).

### Cell cycle and proliferation analysis

HaCaT cells were seeded into 60-mm culture dishes (1 × 10^8^ cells/dish). After incubation for 12 h, cells were exposed to various concentrations of rhein (25 and 50 μM) for 24 h. For proliferation assays, cells were labelled with BrdU (10 μM) for 1 h using the BrdU Flow Kit (BD Science, USA). Cells were fixed and permeabilized with Cytofix/Cytoperm buffer, and cells were then stained using DNase (300 μg/mL) staining buffer. For cell cycle assays, cells were stained with 7-Aminoactinomycin D (BD Science, USA). The cell cycle and proliferation were analysed by flow cytometry (Beckman Coulter, USA).

### Quantitative real-time polymerase chain reaction

After co-culture with 50 μM rhein for 3 h, HaCaT cells were washed with sterilized phosphate buffered saline (PBS) and harvested from 6-well plates using 300 *μ*L TRIzol reagent (Thermo Fisher Scientific Inc, U.S.) per well. The suspension was collected and transferred to 1.5 mL RNase-free EP tubes. Chloroform was then added to inhibit RNase activities and remove phenol. It took 20 min for the mixture to fully react in the room temperature. Subsequently, samples were concentrated in a pre-cooling centrifuge at 4 ℃, 12,000 rounds per minute (RPM) for 10 min. The obtained water phase was transferred to another RNase-free EP tube pre-placed on ice. Equal volumes of isopropanol, 75% ice ethanol, and DEPC water solution were added to measure the OD value and estimate the purity of RNA. Samples with A260/A280 values between 1.8 and 2.0 were used for reverse transcription. The Reverse Transcription Kit (Takara Bio, Japan) was used with a 10 *μ*L reaction volume. Quantitative real-time polymerase chain reaction (qRT–PCR) was performed using a PowerUp SYBR Green Master Mix Kit (Thermo Fisher Scientific Inc, U.S.) on a Thermo Fisher Scientific Real-Time System. GAPDH was amplified as an internal control. The following primer sequences were used: c-myc F, CGAGGAGAATGTCAAGAGGCGAAC; c-myc R, TCGGCGAACTCCTGCTCCTC; FosB F, TCTGTCTTCGGTGGACTCCTTCG; FosB R, TGGAGGTCCTGGCTGGTTGTG; JunD F, CGCCTCATCATCCAGTCCAACG; JunD R, GCTTGGACGGACAGGATGTATGC; GAPDH F, AGAAGGCTGGGGCTCATTTG; and GAPDH R, AGGGGCCATCCACAGTCTTC.

### Western blotting

After co-culture with 50 μM rhein for 24 h, HaCaT cells were washed with sterilized phosphate buffered saline (PBS) and harvested from 6-well plates using 50 *μ*L RIPA lysis buffer (Beyotime, China) per well. And BCA assay was performed to measure the protein concentration with BCA protein analysis kit (Thermo Fisher Scientific Inc, U.S.). For each blotting process, a total amount of 10 μg protein per sample was separated and transferred to a polyvinylidene fluoride membrane. The membranes were then incubated with the following primary antibodies overnight at 4 °C: anti-c-myc antibody (Cell Signaling Technology, 5605S, 1:1000), anti-FosB antibody (Cell Signaling Technology, 2251S, 1:1000), anti-JunD antibody (Cell Signaling Technology, 5000S, 1:1000), and GAPDH antibody (Cell Signaling Technology, 2118S, 1:5000). The membranes were then incubated with appropriate secondary antibodies (MedChemExpress, 7074S, 1:10,000) for 1 h at room temperature.

### Statistical analysis

All results were expressed as mean ± SEM. Statistical analysis was performed using GraphPad Prism 8.0. and Excel 2019. Datum was analysed for significant differences using one-way analysis of variance (ANOVA) and Student’s t test. Differences were considered significant at *P* < 0.05.

## Results

### Binding validation

**Fig. 1:**
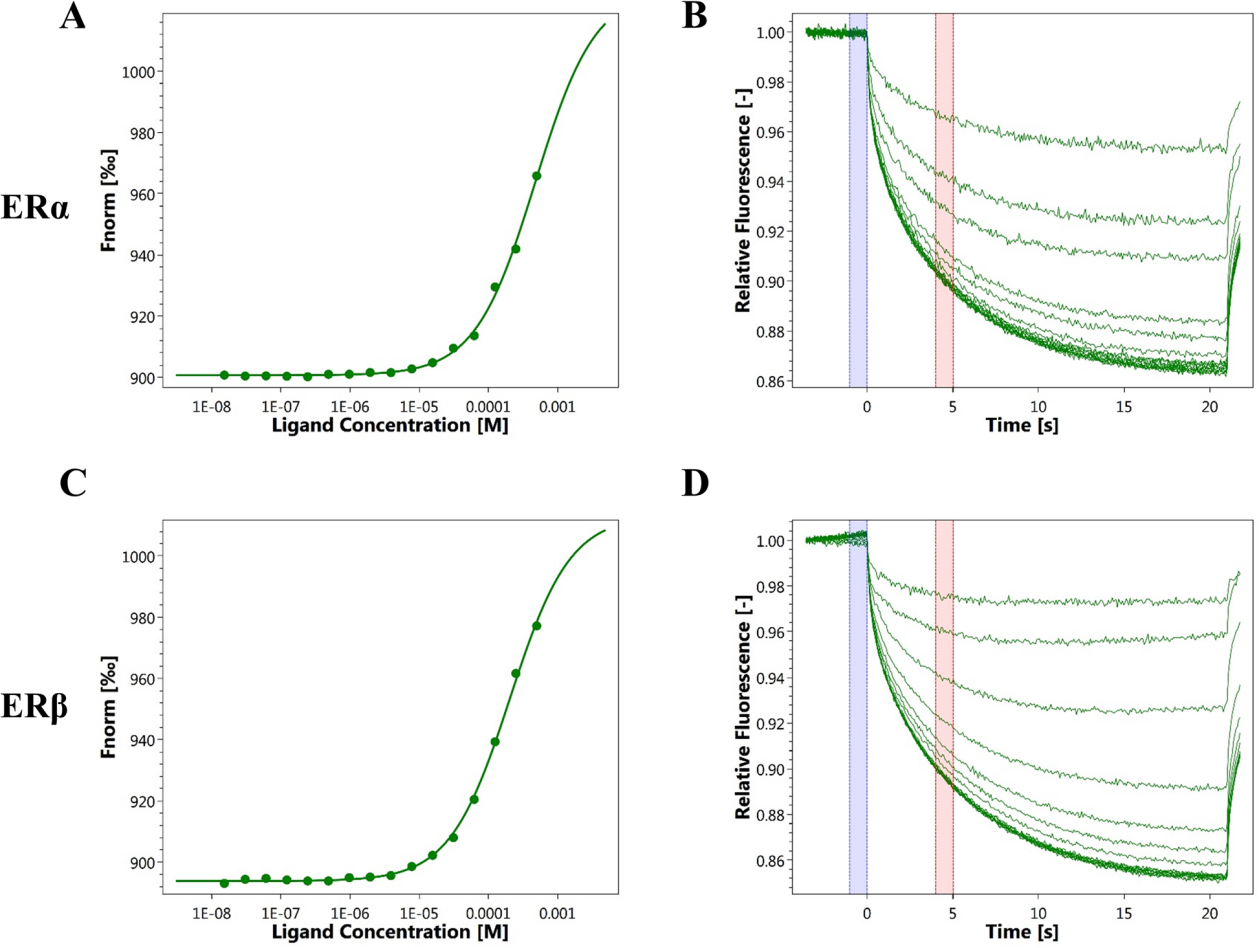
Direct binding ability of rhodopsin to target protein (ERs). Quantification of the binding affinity of rhein to ERs using a microscale thermophoresis assay. **A** and **B** The binding affinity of rhein for ERα (shown in the upper panel). **C** and **D** The binding affinity of rhein for ERβ (shown in the lower panel)

The potential target of rhein was investigated using a computer model-assisted molecular docking technique, and the binding target of rhein was discovered to be the oestrogen receptor. We performed MST assays to examine whether rhein binds to ERs. The results of the MST assays showed that the binding affinity of PA to ERα was 407.01 ± 75.97 μM and that the binding affinity of PA to ERβ was 289.44 ± 18.59 μM, suggesting that rhein maintains specific binding to ERs (Fig. 1).

### Cell viability and proliferation

**Fig. 2:**
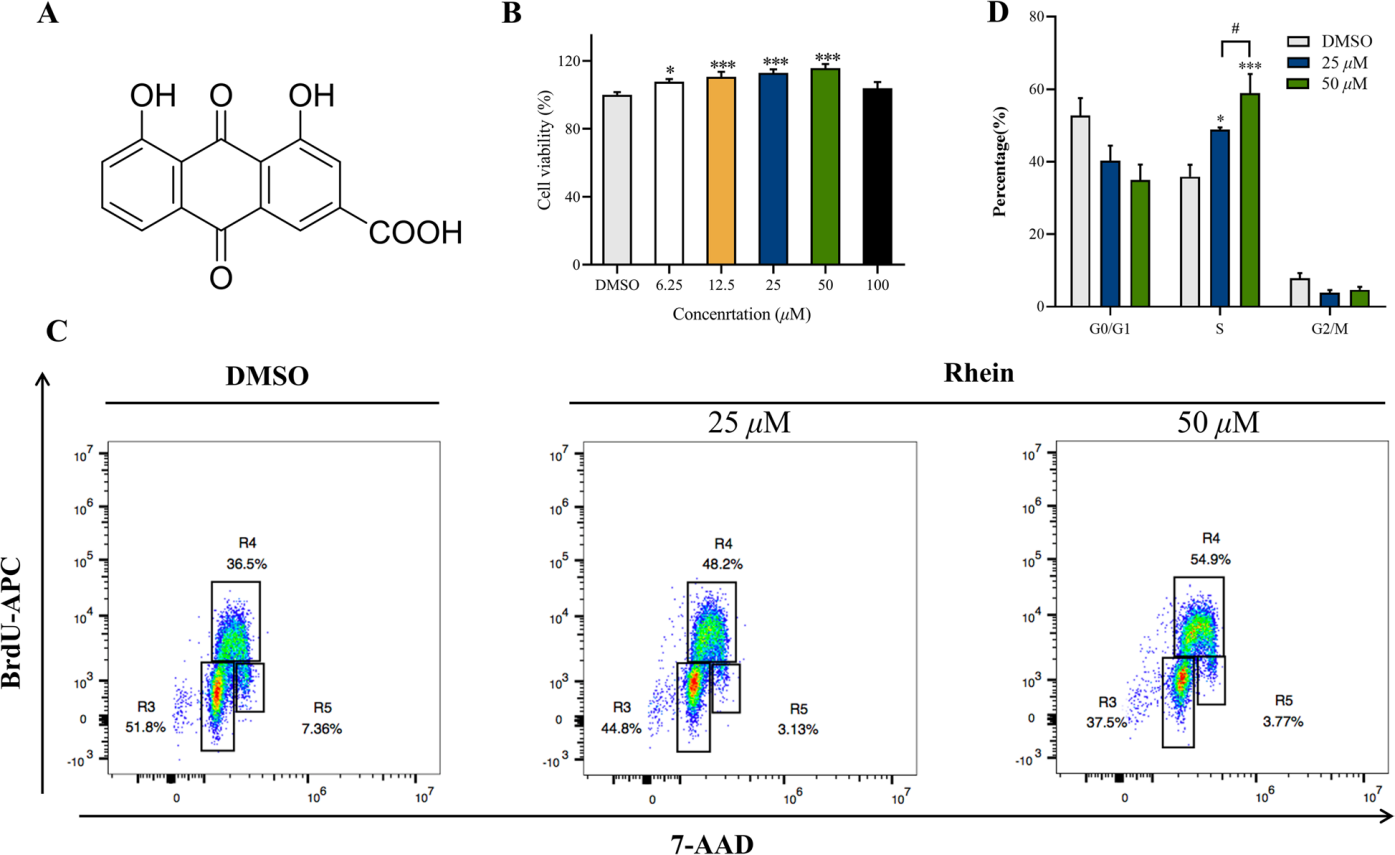
Effects of Rhein on the viability and proliferation of keratinocytes. **A** Structure of rhein. **B** Cell viability of keratinocytes was tested using the CCK-8 assay. **C** Rhein-treated HaCaT cells was labeled with BrdU, and measured by flow cytometry and staining with anti-BrdU antibody. Region 3 (R3) is G_0_/G_1_, region 4 (R4) is S, and region 5 (R5) is G_2_/M. The representative scatter diagrams of each group (DMSO group, Rhein 25 μM group and Rhein 50 μM group) are shown. **D** The results of cell cycle were analyzed by Flowjo version 10.0.7 software. Data are presented as the mean ± SEM. ^*^P < 0.05, ^**^P < 0.01, and ^***^P < 0.001 vs DMSO group; ^#^*P* < 0.05, ^##^*P* < 0.01, and ^###^*P* < 0.001 vs the 25 μM rhein group

To confirm the effects of rhein (Fig. 2A) on the viability and proliferation of keratinocytes, HaCaT cells were incubated with rhein. Intervention of 100 μM rhein for 24 h didn’t reduce the HaCaT viability, while rhein significantly increased the viability of HaCaT cells at concentrations ranging from 6.25 to 50 μM after 24 h compared to the DMSO control (Fig. 2B). We next determined whether rhein might affect the cell cycle of HaCaT cells with APC BrdU flow cytometry. Rhein increased the proportion of S phase cells in the cell cycle and promoted keratinocyte proliferation at concentrations of 25 μM and 50 μM (Fig. 2C and D), and the effect was more significant at a concentration of 50 μM (*P* < 0.05).

### Effects of rhein on estrogen signaling pathway and c-myc level

Then we checked the downstream oestrogen signaling pathway at both mRNA and protein levels.

**Fig. 3:**
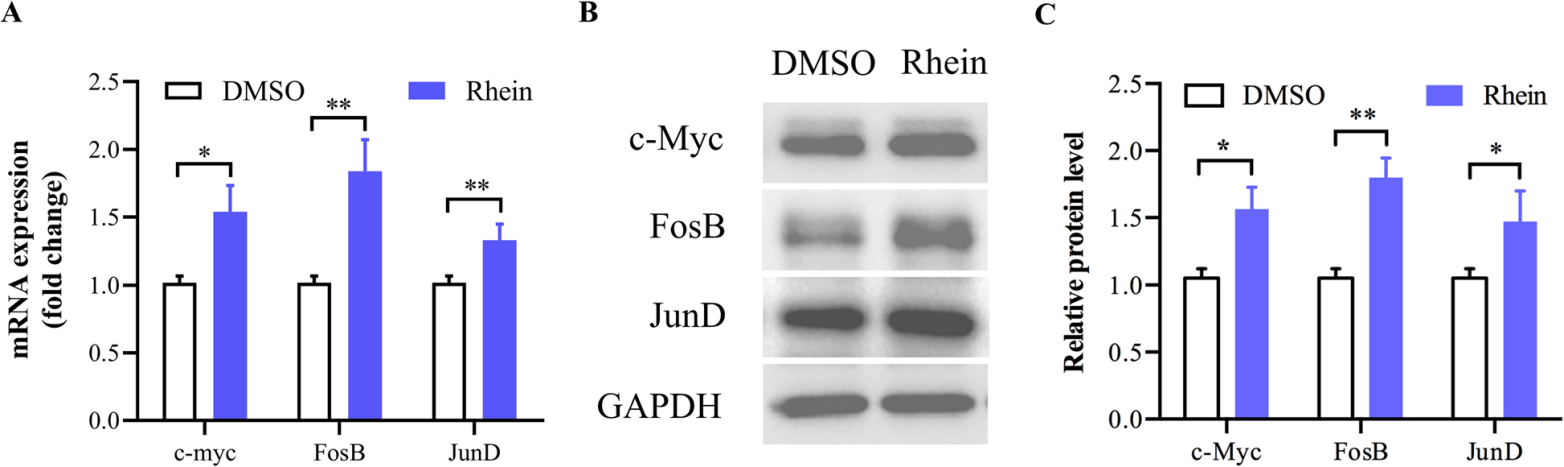
Effects of rhein on estrogen signaling pathway and c-myc expression in HaCaT. **A** mRNA level of *c-myc*, *FosB* and *JunD*. **B** and **C** Protein levels of c-myc, FosB and JunD Full-length blots are shown in Supplementary Fig. S1. Data are presented as the mean ± SEM. ^*^P < 0.05 and ^**^P < 0.01 versus the DMSO group

**Fig. 4:**
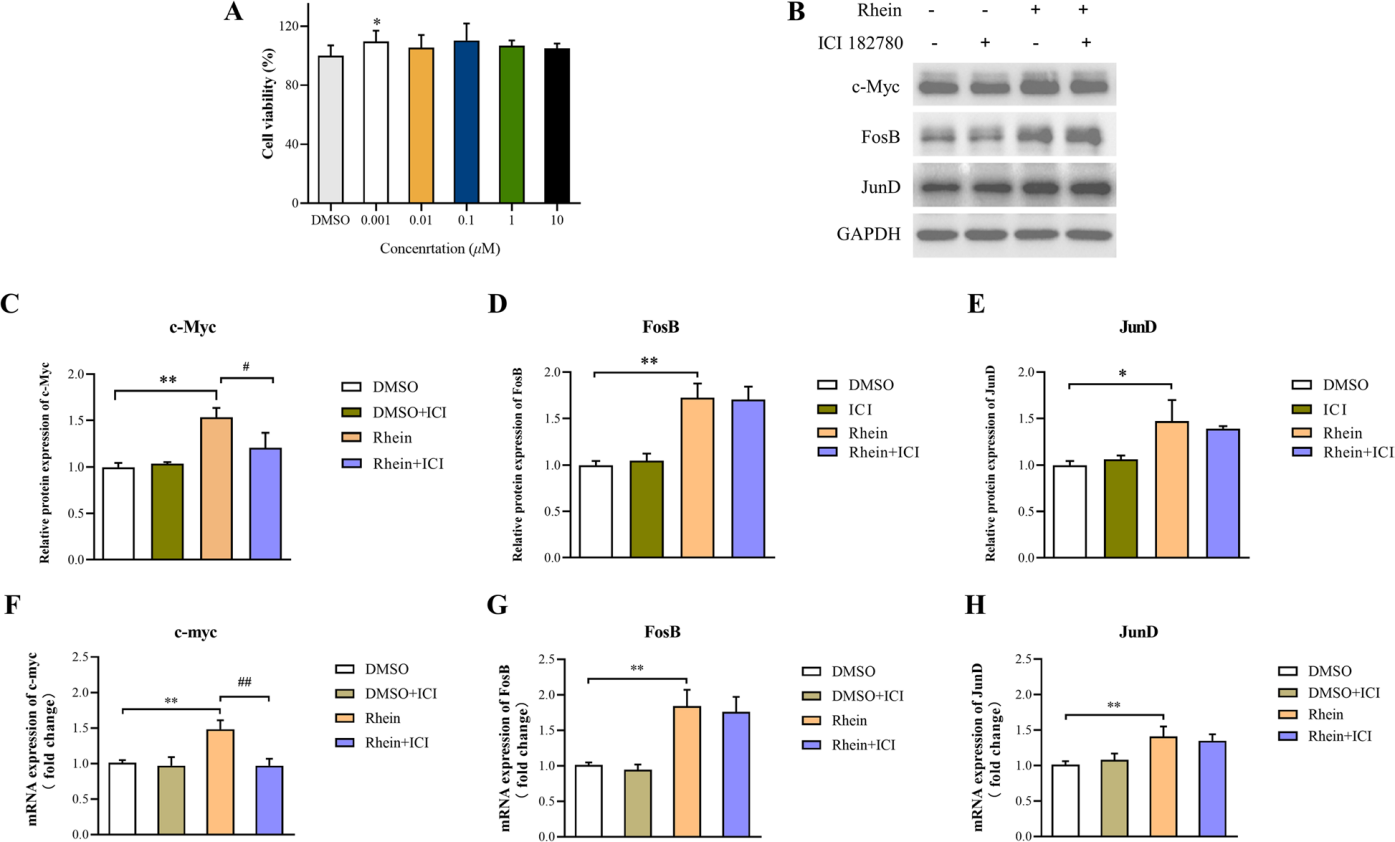
Effects of Rhein on estrogen signaling pathway and c-myc expression under the intervention of estrogen antagonists in HaCaT. **A** cytotoxicity of Estrogen antagonist (ICI 182,780) were measured by CCK-8 assays. **B–E** C-myc, FosB, and JunD protein expression in HaCaT with rhein at 50 μM concentration. **F–H** The mRNA levels of *c-myc*, *FosB*, and *JunD* in HaCaT with rhein at 50 μM concentration. Full-length blots are shown in Supplementary Fig. S1. Data are presented as the mean ± SEM. ^*^P < 0.05 and ^**^P < 0.01 versus the DMSO group, ^##^*P* < 0.01 versus the rhein group

The mRNA expression of c-myc, FosB, and JunD in HaCaT cells were higher in the rhein group compared to the DMSO group (Fig. 3A). Compared to the DMSO group, c-myc, FosB, and JunD were enhanced significantly in the rhein group (Fig. 3B–C). To further explore the mechanism by which rhein regulates c-myc in HaCaT cells, we utilized an oestrogen receptor inhibitor (ICI 182,780). The oestrogen receptor inhibitor impoved HaCaT proliferation insignificantly at all concentrations (*P* > 0.05) but 0.001 μM (Fig. 4A). CCK-8 results of 1 μM and 10 μM groups were much closer to DMSO group, so taking the official manual of ICI 182,780 and former published research [11] into consideration, we used 1 μM for subsequent experiments. The upregulation of c-myc was blocked by the oestrogen receptor inhibitor (*P* < 0.05), but had no significant effect on the protein (Fig. 4B–E) and mRNA (Fig. 4F–H) expression levels of FosB and JunD (*P* > 0.05). These results indicated that rhein activates the oestrogen signalling pathway and cooperates with FosB and JunD to induce the levels of c-myc.

## Discussion

Skin ulcers have diverse etiologies and complex pathogenesis. The chronic inflammatory microenvironment formed by the persistence of inflammatory factors further hinders wound healing and is often closely associated with the majority of persistent ulcers [12]. The formation of skin ulcers disrupts the continuity and integrity of skin tissue, and re-epithelialization is a key component of wound healing and re-establishing tissue integrity [13]. Keratinocytes, as effector cells for re-epithelialization, proliferate and migrate at the trauma surface and play a key role in promoting the restoration of the skin barrier between the internal and external environments [14]. Keratinocytes proliferate, migrate and differentiate to rebuild the skin barrier and participating in epithelialization. Therefore, protecting the function of keratinocytes is important to wound healing [15].

The pharmacological effects of rhein and its derivatives have received more and more attention in scientific research and clinical treatment, covering a wide range of aspects such as liver protection, kidney protection, anti-inflammatory, antioxidant, antibacterial, antitumor, and regulation of glycolipid metabolism [16–20]. It has become one of the hot spots in anti-tumor research because of its significant anti-proliferative and pro-apoptotic effects in a variety of tumor cells through different pathways [21–24]. Therefore, previous studies have focused on the prominent anti-proliferative and pro-apoptotic ability of rhein, and studies on the effects of rhodopsin on the cell cycle have also focused on its blocking effects, while few studies have reported that rhein promotes DNA synthesis and affects the cell cycle by increasing the proportion of S-phase cells in the cell cycle. To confirm the effects of rhein on the viability and proliferation of keratinocytes, HaCaT cells were incubated with rhein. We found that rhein significantly increased the viability of HaCaT cells at concentrations ranging from 6.25 to 50 μM after 24 h. We next investigated the effect on cell cycle using APC BrdU flow cytometry and we found that rhein increased the proportion of S phase cells in the cell cycle and promoted keratinocyte proliferation at concentrations of 25 μM and 50 μM, and the effect was more significant at a concentration of 50 μM. The above experiments confirmed the pro-proliferative effect of rhein on HaCaT cells. It is suggested that rhein may induce the proliferation of keratinocytes by increasing the proportion of S-phase cells in the cell cycle and accelerate the re-epithelialization, thus promoting the healing of skin ulcer wounds.

It has been established that the skin is a hormone-sensitive organ [25]. Exogenous oestrogen treatment may reverse the effects of oestrogen deficiency on wound healing, particularly during the stages of inflammation and tissue remodelling [26]. In addition, clinical practice has discovered that the healing rate of acute skin injury in elderly male patients is significantly slower than that of female patients of the same age and that elderly males more easily develop chronic skin ulcers. The wound-healing ability of postmenopausal women is significantly improved after receiving oestrogen replacement therapy [27–29].

The ER is highly expressed in various cells of the epidermis, dermis, and blood vessels, indicating that oestrogen has an action target in the skin [30]. The oestrogen receptor system is involved in the entire skin defect healing process, influencing granulation tissue formation, re-epithelialization, and remodelling after healing [31]. Oestrogen passes through the cell membrane and interacts with nuclear ERα, and ERβ binding leads to changes in its conformational structure. The complex then translocates into the nucleus and binds to oestrogen receptor elements (EREs) in the promoter region of the target gene to activate or inhibit gene transcription, which is the classical oestrogen signalling pathway [32]. Many oestrogen-regulated genes, such as c-myc, have no ERE in their promoters. Oestrogen regulates gene expression in these genes via other mechanisms. Recent research has indicated that FosB and JunD, which are ER and AP-1 members, do not bind to the distal enhancer region in the absence of oestrogen, and c-myc is expressed at a low level. When oestrogen is introduced into cells, the ER-JunD-FosB complex forms to act as a transactivator, which interacts with the c-myc promoter region, recruits RNA polymerase II (Pol II), and finally induces c-myc gene expression [33]. Thus, oestrogen can also induce c-myc gene expression via its receptor and the AP-1 members, FosB and JunD.

Although c-myc is a member of the proto-oncogene myc family, its role is not limited to tumorigenesis. c-myc is involved in a variety of physiological processes, such as cell growth, proliferation, and differentiation, and it encodes receptors, growth factors, protein kinases, signal molecules, and transcriptional regulators [34]. Interestingly, studies have shown that there is a subtle internal relationship between the occurrence and development of tumours and the repair and healing of wounds [35–38]. In short, the intersection of the two processes covers the following stages: inflammatory response, cell proliferation, differentiation, angiogenesis, and tissue remodelling. Based on these similarities, the overlap on the material basis is not accidental; that is, some key genes, proteins, and even signal pathways not only stimulate tumour formation but also participate in the corresponding process in wound healing [39]. Thus, c-myc is important not only in skin wound repair and healing but also in tumour transformation. However, the distinction is that the outcomes are diametrically opposed. Wound healing is relatively conservative and controllable in comparison to the absolute uncontrollable excess of tumour growth and infiltration. External intervention and stimulation induce c-myc expression and promote the cell cycle from G0/G1 to S phase, resulting in cell division and proliferation [40]. Although cell proliferation is not good when combating tumours, cell proliferation is necessary for wound healing. Furthermore, the upregulation of c-myc expression during the healing process of skin ulcers can be regarded as a proliferation marker of skin cells at a certain level.

Previous microarray results have highlighted the significant upregulation of the c-myc gene after SJHY intervention in HaCaT cells. In vitro experiments also confirmed that SJHY has a proliferation-promoting effect on HaCaT cells at a specific concentration of 10 mg/L, and this effect is accompanied by an increase in c-myc protein expression. However, SJHY has no effect on the phosphorylation level of c-myc, suggesting that the increase in its expression level is regulated at the level of translation and transcription. However, it remains unknown how SJHY regulates the expression of c-myc.

In the present study, we confirmed the direct binding ability of rhein to ERα and ERβ using MST technique, which provides direct evidence to investigate the molecular pharmacological mechanism of rhein, suggesting that rhein exerts estrogen-like effects via estrogen receptors during skin ulcer healing. Further experiments confirmed that rhein interferes with HaCaT cells for 24 h, promotes cell proliferation by increasing the proportion of S-phase cells in the cell cycle, and facilitates the promotion of wound healing through re-epithelialization. Previous studies have shown that estrogen rapidly and stably induces c-myc expression in estrogen receptor-positive breast cancer cells (MCF-7). c-myc mRNA induction occurs as early as 30 min and persists for 6 h after treatment [33]. As a result, we treated HaCaT cells with rhein for 3 h, resulting in increased mRNA expression levels of c-myc, FosB,and JunD. 24 h later, rhein increased the protein expression of c-myc, FosB, and JunD in HaCaT cells. The endoplasmic reticulum inhibitor ICI 182,780 was used in treated cells to study the endoplasmic reticulum dependence of c-myc expression. Rhein-induced c-myc mRNA and protein expression was antagonized by ICI 182,780, indicating that this induction is endoplasmic reticulum-dependent. ICI 182,780 intervention had no effect on the upregulation of FosB and JunD, indicating that they are involved in rhein-induced c-myc mRNA and protein expression, but are not required for ER.

## Conclusion

**Fig. 5:**
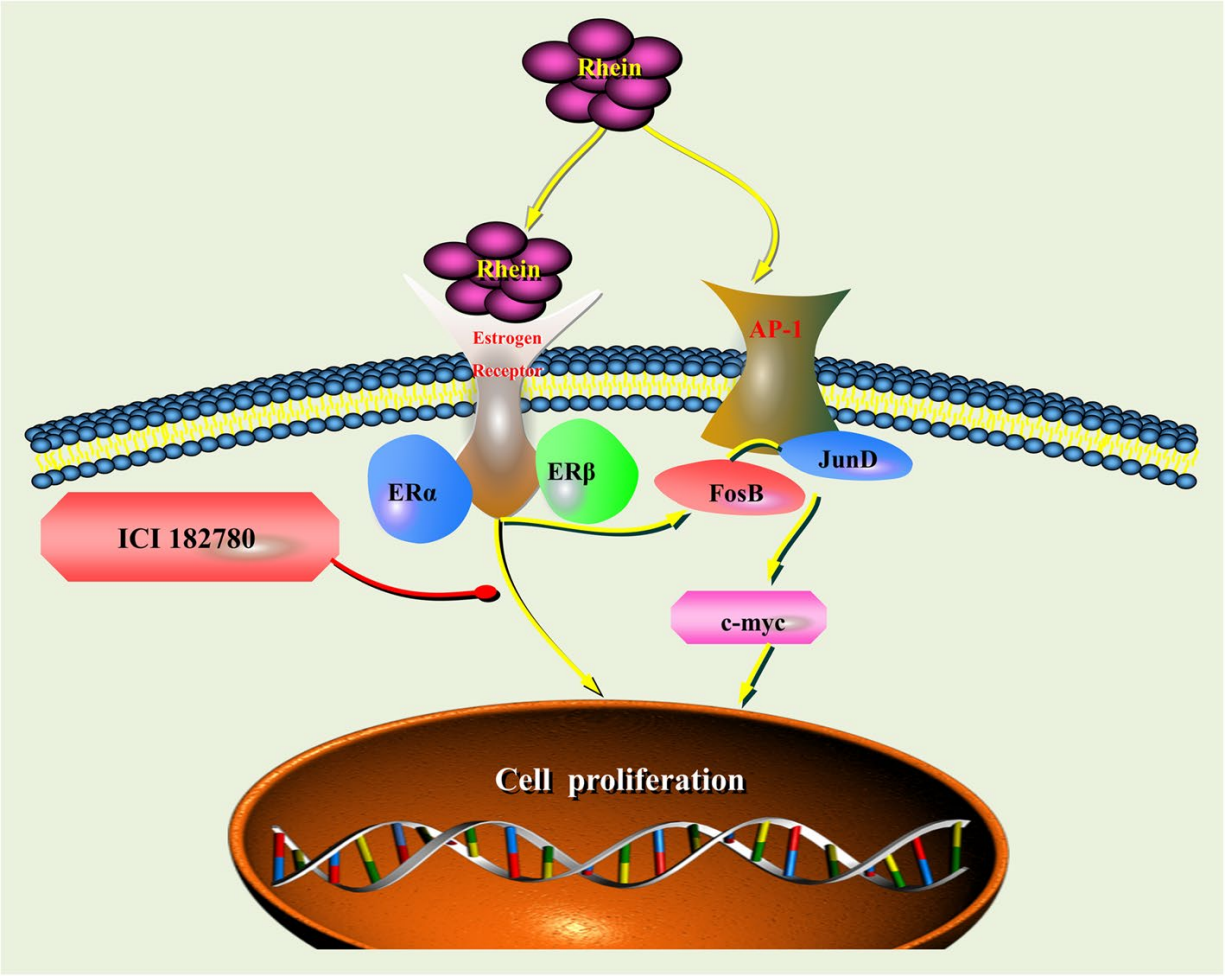
Schematic illustration of the underlying mechanism of the oestrogenic activity of rhein via the oestrogen receptor in keratinocytes

In conclusion, our findings showed that rhein activates the oestrogen signalling pathway via the oestrogen receptor, induces c-myc expression and promotes keratinocyte proliferation, thereby accelerating the process of re-epithelialization (Fig. 5).

## Supplementary Information

**Additional file 1:** **Supplementary Figure S1.** Full-length blots from different gels showing the expression levels of c-myc, FosB, JunD, and GAPDH.

## Data Availability

The datasets supporting the conclusions of this article are included within the article.

## Abbreviations

SJHY: Sheng-ji Hua-yu
TCM: Traditional Chinese Medicine
MST: Microscale Thermophoresis
ER: Oestrogen Receptors
RT-PCR: Real-time polymerase chain reaction
AP-1: Activator protein 1
ERE: Oestrogen Receptor Elements
Pol II: RNA polymerase II
