# Supplementary material for: Rhein promotes the proliferation of keratinocytes by targeting oestrogen receptors for skin ulcer treatment
Source: BMC Complement Med Ther. 2022 Aug 5;22:209. doi: 10.1186/s12906-022-03691-1 (PMC9354312; doi:10.1186/s12906-022-03691-1)
Supplement: Supplementary file 1 — Additional file 1: Supplementary Figure S1. Full-length blots from different gels showing the expression levels of c-myc, FosB, JunD, and GAPDH. [file 12906_2022_3691_MOESM1_ESM.docx]

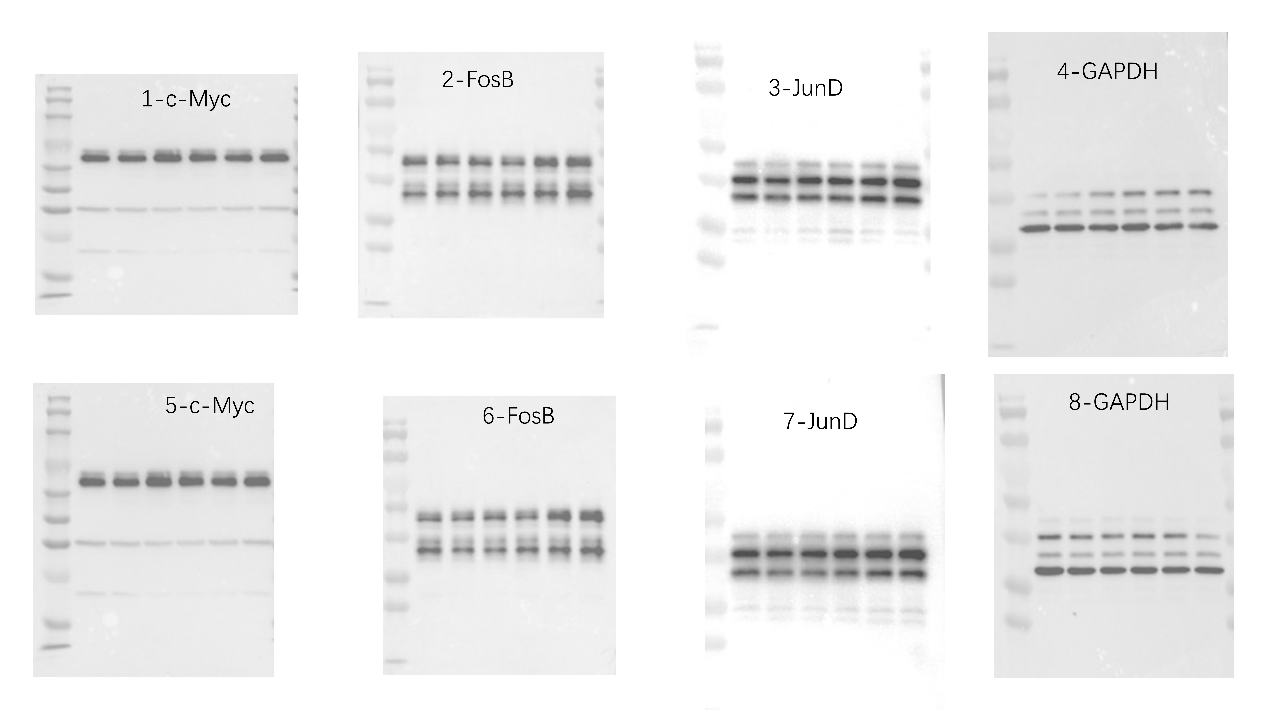


**Supplementary Figure S1**. Full-length blots from different gels showing the expression levels of c-myc, FosB, JunD, and GAPDH.
